# Practical Recommendations for the Management of Benign Adnexal Masses

**DOI:** 10.1055/s-0040-1714049

**Published:** 2020-06-19

**Authors:** Rodrigo Manieri Rocha, Ionara Diniz Evangelista Santos Barcelos

**Affiliations:** 1 Departament of Gynecology and Obstetrics, Universidade Estadual do Oeste do Paraná, Cascavel, Paraná, PR, Brazil

**Keywords:** adnexal masses, ovarian cysts, ovarian surgery, ovarian reserve, infertility, fertility preservation, massas anexiais, cistos ovarianos, cirurgia ovariana, reserva ovariana, infertilidade, preservação da fertilidade

## Abstract

**Objective** To perform a comprehensive review to provide practical recommendations regarding the diagnosis and treatment of benign adnexal masses, as well as information for appropriate consent, regarding possible loss of the ovarian reserve.

**Methods** A comprehensive review of the literature was performed to identify the most relevant data about this subject.

**Results** In total, 48 studies addressed the necessary aspects of the review, and we described their epidemiology, diagnoses, treatment options with detailed techniques, and perspectives regarding future fertility.

**Conclusions** Adnexal masses are extremely common. The application of diagnosis algorithms is mandatory to exclude malignancy. A great number of cases can be managed with surveillance. Surgery, when necessary, should be performed with adequate techniques. However, even in the hands of experienced surgeons, there is a significant decrease in ovarian reserves, especially in cases of endometriomas. There is an evident necessity of studies that focus on the long-term impact on fertility.

## Resumo

**Objetivo** Realizar uma revisão abrangente para fornecer recomendações práticas sobre o diagnóstico e tratamento de massas anexiais benignas, bem como informações para um consentimento adequado com relação à possível perda da reserva ovariana.

**Métodos** Uma revisão abrangente da literatura foi realizada para identificar os dados mais relevantes sobre o assunto.

**Resultados** No total, 48 estudos abordaram os aspectos necessários da revisão, e descrevemos sua epidemiologia, diagnósticos, opções de tratamento com técnicas detalhadas, e perspectivas sobre fertilidade futura.

**Conclusões** As massas anexiais são extremamente comuns. A aplicação de algoritmos de diagnóstico é obrigatória para excluir a malignidade. A maioria dos casos pode ser manejada conservadoramente. A cirurgia, quando necessária, deve ser realizada com técnicas adequadas. No entanto, mesmo nas mãos de cirurgiões experientes, há diminuição significativa da reserva ovariana, principalmente nos casos de endometriomas. Há uma evidente necessidade de estudos que enfoquem o impacto das massas anexiais benignas na fertilidade em longo prazo.

## Introduction

Benign adnexal masses are extremely common during a woman's life. Up to 10% of them will undergo surgery due to some adnexal mass throughout their lifetime. 1 Because it is a benign disease, the surgical treatment may seem deceptively simple. How can we properly inform patients who are concerned about fertility?

Despite the high prevalence of this condition and the number of surgical procedures, a significant part of the patients does not seem to receive enough information for appropriate consent regarding possible loss of the ovarian reserve. 2 There is evidence of negative impact on ovarian reserve due exclusively to the presence of cysts 3 4 and perhaps more markedly in endometriotic cysts. 5 In addition, a considerable proportion of these patients will undergo surgery that could potentially compromise even more the ovarian function. 6

It is extremely important that the attending physician has a critical view of the difficulties in assessing adnexal masses, the consequences of the clinical and surgical treatments, and the current possibilities of gauging the ovarian reserve, since the treatments and the different techniques may interfere on fertility in the long term.

The objective of the present review is to provide the gynecological surgeon with fundamental elements for a better understanding of the decision-making process in the treatment of adnexal pathologies and the best possible counseling for women about the risks and benefits of these treatments in their reproductive future. The establishment of appropriate surgical routines that consider the preservation of fertility is of major importance.

## Methods

The data were obtained through a bibliographic research in the Medline, LILACS and Scielo databases. We performed the search using the terms “ *ovarian cyst* ,” “ *adnexal mass* ,” “ *ovarian reserve* ,” “ *ovarian surgery* ,” “ *infertility* ” and “ *fertility preservation* .” All identified articles were published in English in the past 15 years (between January 2004 and April 2019). The articles that had as their central topic benign ovarian pathologies and their relationship with alterations in the ovarian reserve and preservation of fertility were included.

## Results

A total of 48 articles published between 2004 and 2019 fulfilled the inclusion criteria. The information was analyzed for the consistency of the data, the year of publication, and the quality of the study. Articles that did not address directly the review objectives were excluded.

## Discussion

### Epidemiology

The ovarian cyst is defined as a structure that contains fluid and has more than 30 mm in diameter. 1 A significant part of the ovarian cysts is asymptomatic, which can lead one to underestimate the real incidence. It is estimated that, in the United States, 250 thousand women per year are hospitalized due to adnexal masses. 7

According to a population-based cohort with 11,595 patients from 1991 to 2014, 8 the incidence of ovarian cysts increases exponentially with age. There is an incidence plateau around the age of 26, reaching 152 cases per 100 thousand women per year at 35 years old, which is maintained throughout the menacme. 8

**Fig. 1:**
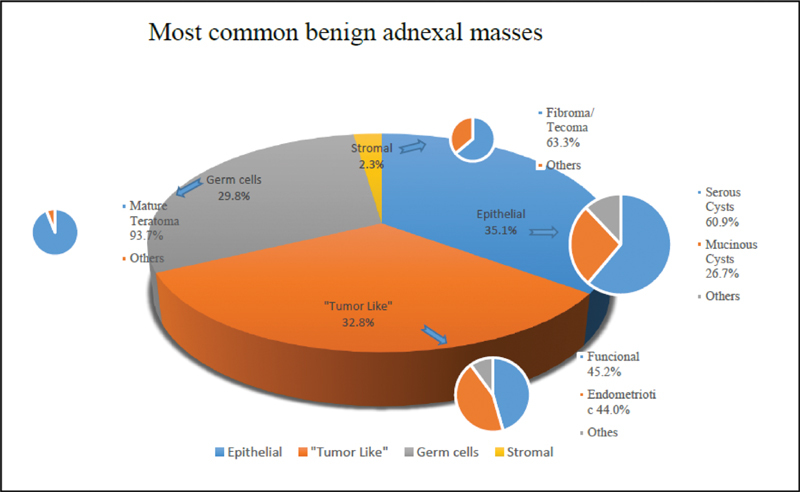
Most common benign adnexal masses.

Just over a third of the benign masses were epithelial lesions (35.1%). Almost a third were tumor like lesions (32.8%), being functional cysts and endometriotic cysts. 29.8% were germ-cell tumors (almost entirely mature teratomas), and a small fraction of 2.3% were stromal tumors (mostly fibromas/tecomas). The exact proportions are shown in Fig. 1 .

At menacme, most of the masses are benign, and the chance of developing malignancy of the symptomatic cysts is around 1: 1 thousand. 1 The incidence of borderline malignancies and tumors follows the same behavior with regard to the incidence plateau; however, it is of around 8 cases per 100 thousand women per year. Therefore, the proportion of malignancies among all ovarian neoplasms is of ∼ 3%. 8 In the pediatric range, the common symptoms are acute and chronic abdominal pain, presence of abdominal mass, and abdominal distension. Germ-cell tumors are more common, with teratomas being the main representatives. Physiological cysts in premenarchal girls are rare, and it is estimated that the prevalence of malignancy does not exceed 15%. 9

### Diagnostics

We can conclude from the previous section that, in fact, the chance of facing malignant adnexal pathologies is relatively low. Despite this, the identification of malignant neoplasms is mandatory. The difficulty in establishing a reasonable means of screening for ovarian malignancies is widely known. Most ovarian cancers are not diagnosed in the early stages (15% in stage I), even in the menopausal period, when the incidence of this type of malignancy increases. 10

Many models have been proposed to screen patients for appropriate treatment, but none of them was able to be unanimously accepted. The details of the various models are beyond the scope of the present review, but they may include: the Risk of Malignancy Index (RMI); the National Institute for Health and Care Excellence (NICE) Guidelines; the Risk of Ovarian Malignancy Algorithm; Logistic Regression (LR); and methods based on ultrasound criteria proposed by the International Ovarian Tumor Analysis (IOTA): “Simple Rules” (SRs); Simple Rules Risk (SRR); and Assessment of Different Neoplasms in the Adnexa (ADNEX). 11

Despite some modifications over the years after the creation of the RMI, it remains as the alternative with the best validation. 1 It consists of the product between the level of carcinoembryonic antigen 125 (CA-125), hormonal status (M) and ultrasound scoring (U): RMI = CA-125 x M x U ( Table 1 ).

**Table 1:** Risk of Malignancy Index (RMI)

RMI CRITERIA	SCORE
MENOPAUSAL STATUS (M) ^*^	
** Premenopausal**	1
** Postmenopausal**	3
**ULTRASOUND IMAGING (U)**	
** Multiloculated**	1 (if 1 feature)
** Solid areas**	3 (if 2 or more features)
** Bilaterality**	
** Ascites**	
**SERUM CA-125**	Absolute value in IU/mL

In order to create an adequate screening workup, the use of a specific set of ultrasound characteristics to predict malignancy of the adnexal masses named the “Simple Rules” (SRs) was recently proposed. 12 The concept is based on the identification of basic benign and malignant echography features ( Box 1 ).

**Box 1:** International Ovarian Tumor Analysis (IOTA) “Simple Rules”

IOTA “SIMPLE RULES”	
Benign (B)	Malign (M)
1- Unilocular cyst	1- Irregular solid tumor
2- Solid component, but < 7 mm	2- Ascites
3- Acoustic shadows	3- At least 4 papillary structures
4- Smooth multilocular tumor < 100 mm	4- Irregular multilocular tumor > 100 mm
5- No blood flow	5- Very strong flow

Eight years later, in 2016, a large multicenter cross-sectional study 13 involving 22 research centers with 4,848 patients aimed to evaluate the efficacy of the SRs in the prediction of malignancy of adnexal masses. For the 1% risk cutoff, the sensitivity was of 99.7%, the specificity was of 33.7%, the LR was + 1.5 and -0.010, the positive predictive value (PPV) was of 44.8%, and the negative predictive value (NPV) was of 98.9%. For the 30% risk cutoff, the sensitivity was of 89.0%, the specificity was of 84.7%, the LR was + 5.8 and -0.13, the PPV was of 75.4%, and the NPV was of 93.9%. 13

The use of SRs was subsequently validated considering their performance in the diagnosis of early ovarian malignancies, even when compared with the other models proposed by the IOTA. At this point, we highlight the high NPV (98.9%) of the SRs in scenarios of low risk of malignancy. 13

After the application of the screening methods, the patients whose initial evaluation reveals high risk for malignancy should ideally be treated in specialized centers with a multidisciplinary team, maximizing the oncological results and making it possible to discuss options to preserve fertility. 14

### Ovarian Reserve

There is currently no ideal method to predict the ovarian reserve, and some important aspects should be considered. Estimates of the ovarian reserve are performed mainly by the measurement of the antimullerian hormone (AMH) and the antral follicle count (AFC). The AMH is produced by granulosa cells from active follicles, and can be dosed in the peripheral blood. The production of AMH is stable throughout the menstrual cycle, and is the first to change with advancing age. The levels of AMH are related to some outcomes of assisted reproductive technology (ART), such as the number and the quality of yielded oocytes and the live-birth rate (LBR). 15 16 The diversity of laboratory kits available for AMH dosage generates distortions and differences in reference values, which can cause bias in the interpretation of results. In addition, since the AMH is produced by both ovaries simultaneously, the actual impact of the surgical procedures may be underestimated in cases of unilateral disease. 17

The AFC consists of the sum of the 2- to 10-mm ovarian follicles found between the 2nd and 4th days of the menstrual cycle, and it is also related to ART outcomes. 17

It has already been proposed that the association of methods (dosage of AMH and AFC) may contribute to reduce the risk of misunderstandings. In the event that the levels of AMH are higher than expected given an AFC, the presence of polycystic ovarian syndrome should be considered. And levels of AMH lower than expected for a given AFC may be an early sign of ovarian failure. 18

Some of the studies mentioned in the present review also used other methods such as the follicle-stimulating hormone (FSH), ovarian volume measurement and ovarian peak systolic velocity (PSV), but they are less reliable. 19

## Treatment

### Surveillance

A significant proportion of the benign ovarian masses will present spontaneous resolution. Therefore, the expelling conduct may be of extreme validity, avoiding unnecessary surgeries, and, consequently, additional damage to healthy ovarian tissue. Functional cysts or simple ovarian cysts (anechogenic, thin-walled and smaller than 50 mm) usually resolve after 3 menstrual cycles without the need for any intervention. 1

### Management of the Risk of Adnexal Torsion

After the decision regarding surveillance, the risk of adnexal torsion remains a concern. In an international prospective cohort, among the patients selected for conservative management and surveillance, the overall risk for torsion was of around 4%. 20

A case series 21 with 360 patients showed that the main pathologies associated were dermoid cysts (36%), follicular cysts (16.1%), corpus luteum cysts (9.9%), and serous cystadenoma (9.9%), with an overall mass size ranging from 8 cm to 15 cm. Functional cysts were successfully treated mainly with detorsion. 21

An interesting proposal in the symptomatic pediatric/adolescent range is a composite score of risk. It consists of identifying and scoring the presence of independent risk factors: absence of vomiting (zero points) or presence of vomiting (2 points); ovarian volume higher than 17 mL (pre-menarchal; 2 points) or 105 mL (menarchal; 2points); adnexal ratio (volume of the affected ovary divided by the contralateral ovary) higher than 21 (2 points). For scores ≥ 4, surgical treatment is recommended. Even with scores between 2 and 3, until 10% of the patients presented with torsion. 22

### Regarding Endometriomas

A study 23 monitored 1,199 cycles of 244 patients with unilateral endometriomas, comparing ovulation between the healthy and the affected ovaries. Documented ovulation did not differ (49.7% versus 50.3% respectively), even with cysts with more than 6 cm in diameter. In total, 43% of the patients conceived spontaneously during the study period of 4 years. 23 There is no relevant data on the literature that supports that systematic surgical removal of cysts smaller than 4 cm prior to assisted reproduction procedures. Expectant behavior is also justified in this scenario. 24 Other aspects regarding the management of endometriomas will be further discussed, but surveillance seems to be the main approach.

### Oral Contraceptives

A systematic review 7 that analyzed eight randomized trials concluded that, although widely used in the clinical practice, the prescription of oral contraceptives does not influence the resolution of functional cysts. The outcomes are the same for both spontaneous cysts and those caused by ovulation-induction processes. Persistent cysts tend to be pathological rather than physiological. 7

### Surgical Approach - Technical Considerations

The indication of surgery occurs when: there is uncertainty regarding the suspicion of malignancy; the size of the adnexal mass increases the risk of pain and torsion episodes; the augmentation of the mass might compromise the ovarian follicles; and, in cases of endometriotic cysts, when there is a progressive increase in volume and pelvic pain due to endometriosis. 25 Once the need for intervention is defined, one should consider several technical details that can minimize the impact on the ovarian reserve and improve the overall outcome of the surgery.

#### Laparoscopy

The body of evidence in favor of the laparoscopic approach is significant when there is a need for surgery. Laparoscopic cystectomy is often the rule, to minimize potential complications such as ruptures in malignant situations, and to optimize fertility preservation. This technique is well established as the gold standard in this set of pathologies. 1 25 26

#### Comparison of Etiologies

Despite the fact that the multitude of etiologies of the ovarian masses and the surgical practice demonstrate evident technical differences among the different types of cysts, the damage to the ovary is evident and unavoidable in every situation.

The comparison of the levels of AMH, FSH, AFC, ovarian volume and vascularization did not show significant differences when comparing the group with bilateral endometriomas ( *n* = 21), unilateral endometriomas ( *n* = 29), other benign cysts ( *n* = 20), and the control group without cysts ( *n* = 20), when the laparoscopic surgery was performed with hemostatic sutures. The small difference observed was in the first postoperative month, but did not remain after 12 months. 27

Comparing the levels of AMH and the amount of remaining follicles in the surgical specimen, no differences were found between patients with endometriomas ( *n* = 68) and other benign cysts ( *n* = 32). Bilaterality was the only risk factor associated with greater loss of ovarian reserve. 28

In a study 29 with 71 patients, the final volume was lower (2.41 mL versus 2.23 mL, *p* = 0.49), as compared with the non-operated ovary, as well as the AFC (3.45 versus 2.43, *p* = 0.11), regardless of the fact that there were endometriotic cysts or not. Ovarian volume and CFA differences were not dependent on the size of the cysts. 29

Contrasting this scenario, in a study 30 comparing the AMH levels of patients with endometriomas, other benign cysts and patients with infertility of tubal cause, endometriomas show a different behavior from the other benign cysts, since AMH levels may be lower than in the other conditions (1.53 ng/mL, 2.20 ng/mL, and 2.82ng / ml respectively). Surgery significantly decreases the levels of AMH in the case of endometriomas (worse if bilateral) compared with other pathologies. 30

In a study 4 with 75 patients (33 teratomas, 25 endometriomas, 9 functional cysts, and 8 cystadenomas) the reduction in the levels of AMH was different, depending on the histological diagnosis of the mass. Patients with endometriomas had a faster and longer lasting reduction in AMH levels: a 51-% mean reduction 6 months after surgery ( *p* = 0.007). Patients with teratomas presented a 25-% reduction ( *p* = 0.009). In the other patients, the reduction was of 34% ( *p* = 0.059). 4

In a study 31 with 22 patients, 12 with endometriomas and 10 with non-endometriotic cysts, there was a reduction in the levels of AMH postsurgery (5.48 ng/mL before and 2.56ng/mL after; *p* < 0.05), but without changes in the AFC, estradiol and ovarian volume. This decrease occurred at the expense of the group of patients with endometriomas. A technique of bipolar hemostasis associated with hemostatic suture was used. 31

A retrospective study 3 of 97 patients aged between 20 and 39 years showed that AMH levels are lower in patients with endometriomas compared with other benign cysts (4.12 ng/mL versus 6.02 ng/mL; *p* < 0.001). The mean level of AMH was also lower the larger the diameter of the non-endometriotic mass. 3

In a case-control study 32 with 56 patients with endometriomas and 16 patients with other benign cysts, there was a significant decrease in AMH levels after surgery (stripping cystectomy with bipolar hemostasis) in the group with endometriosis (4.3 ng/mL versus 2.8 ng/mL; *p* < 0.001), but the same did not occur in the group of other benign cysts (5.6 ng/mL versus 4.9 ng/mL; *p* = 0.251). This drop was also more significant in the group with endometriosis of stages III to IV (4.26 ng/mL versus 2.62 ng/mL; *p* < 0.001) when compared with the group with endometriosis of stages I to II (4.38 ng/mL versus 3.34 ng/mL; *p* = 0.66). 32

Another retrospective study 33 with 138 women submitted to salpingectomy, 36 submitted to unilateral salpingo-oophorectomy, 40 who underwent excision of an endometrioma, and 41 who underwent cystectomy due to other causes showed no difference in the levels of AMH ( *p* = 0.33), AFC ( *p* = 0.59) and FSH ( *p* = 0.21) between the salpingectomy group and the group who did not undergo surgery. The group submitted to unilateral salpingo-oophorectomy had lower levels of AMH (-54%; *p* = 0.001). Women with endometrioma also had lower levels of AMH (-66%; *p* = 0.002), but this did not affect the AFC ( *p* = 0.22) and FSH ( *p* = 0.28). 33

Recently, a study performed in patients 34 with endometriomas ( *n* = 34) and other benign masses ( *n* = 18) showed that, 6 months after surgery, the levels of AMH were reduced by 59.3% ( *p* < 0.12) when compared with baseline values in the group with endometriomas, and it was reduced by 29.5% ( *p* < 0.2) in the group with other benign masses. This reduction was not related to the number of follicles inadvertently removed during the procedure ( *p* < 0.669). It is very important to note that, in this study, all procedures were performed by a single specialist surgeon, which indicates that postoperative damage to the levels of AMH is evident, even for surgeons with extensive experience. 34

In addition, a retrospective study 35 revealed that there are no differences in ovarian stimulation response (measured through retrieved oocytes) in in vitro fertilization (IVF) cycles when there is an evident ultrasound diagnosis of dermoid cyst. 35

#### Endometriomas - Options of Surgical Techniques and Hemostasis

Several years ago, some advantages were associated with the excision of an endometriotic cyst when compared with several types of ablative processes. There was an assumption that the excision of the cyst capsule was associated with a lower recurrence of pain symptoms (dysmenorrhea, dyspareunia and acyclic pelvic pain) and less need for further surgeries. In addition, it was associated with a higher rate of spontaneous gestation after the procedure. It was still unclear whether excision was superior to ablative procedures for ART outcomes. 26

In 2011, a randomized study 36 performed with 48 patients with bilateral endometriomas compared cystectomy and coagulation and found, after cystectomy, a lower antral follicle count (3.67 versus 4.75; *p* = 0.001), lower ovarian volume (6.27 mL versus 9.87 mL; *p* = 0.005) and fewer oocytes collected after ovarian hyperstimulation (3.08 versus 3.86; p 0.01) compared with coagulation alone. 36

In a study 37 with 25 patients with endometriomas, using only stripping, without coagulation, there was no difference in presurgical AMH levels and 3 cycles after surgery (3.61 ng/mL versus 3.00 ng/mL respectively; *p* = 0.62). 37

In another study 38 with 99 patients who underwent surgery for endometriomas, the comparison between cystectomy and bipolar vaporization showed a decrease of more than 50% in AMH levels after surgery. There was no difference between the techniques. 38 It is imperative, however, to highlight some limitations of the mentioned study. It was not randomized, did not make a clear separation of the groups, and did not present any follow-up data.

In yet another study, 39 45 patients with unilateral endometriomas were treated with laparoscopy and cystectomy with striping and hemostasis with a dual-wavelength laser (Biolitec Ceralas HPD, wavelength of 980 nm and 1470 nm, model 120 W). The mean level of AMH before surgery was of 3.01 ng/mL; 4 to 6 weeks after surgery, it was of 2.41 ng/mL; and 6 to 9 months after, it was of 2.7 ng/mL. The decrease was statistically significant ( *p* < 0.05). 39 However, the technique was not compared with stripping alone or with other techniques.

The use of hemostatic sealants was compared with bipolar coagulation in a non-randomized study. 40 The rate of decline in AMH levels was lower using the sealants (15.4%) compared with bipolar coagulation (41.2%; *p* = 0.003). 40 The levels were measured in 129 patients, without randomization, a follow-up of only 3 months, and other ovarian reserve parameters were not measured.

In a more recent study, 41 207 patients who underwent excision of endometriomas were followed up for 12 months. The levels of FSH, AMH, the AFC and the PSV were compared regarding 3 different hemostasis techniques: bipolar cauterization ( *n* = 69), ultrasonic scalpel ( *n* = 69) and suture ( *n* = 69). Throughout the period up to the 12th month, the levels of FSH were higher, and the levels of AMH were lower, in the first 2 groups ( *p* < 0.05). At the 12th month, the AFC and PSV were also lower in the first 2 groups ( *p* < 0.05). The authors concluded that bipolar cauterization and the use of ultrasonic scalpel cause more damages to the ovaries when compared with hemostatic suture. 41

Recently, the laparoscopic stripping of endometriotic cysts became the standard procedure, since it favors a lower recurrence of symptoms and increases pregnancy rates. 42

Despite this, one cannot deny that surgical damage to ovarian tissue is evident. The histological analysis of the endometrioma capsules revealed the presence of normal ovarian follicles, in a larger quantity the younger the patient and the smaller the cyst diameter. 43

Those analyses suggest that the intervention leads to the decrease in the ovarian reserve, although the literature is controversial and heterogeneous, especially considering the different methods of hemostasis. 44 45 The evidence points to a tendency to believe that suture is less harmful to the ovarian reserve.

#### Additional Relevant Technical Details

Following cystectomy, removal of ovarian tissues from the abdomen should preferably be performed through the umbilical portal and wrapped in a protective pouch. This decreases the chance of eventual contamination of the abdominal cavity with the contents of the cyst, and causes less postoperative pain and a shorter recovery time. Transverse minilaparotomy should be considered for the cases of masses with a larger volume (higher than 7 cm in diameter). 1

The phase of the menstrual cycle for the surgery does not seem to influence the results regarding blood loss and variations in AMH levels. In yet another study, 46 84 patients in the follicular phase and 71 in the luteal phase were compared after cystectomy for surgical blood loss ( *p* = 0.984) and AMH levels before and 3 months after surgery ( *p* = 0.945); no differences were found by the authors. 46

During the surgical approach, one should take into consideration the anatomical position of the vascularization of the ovary, aiming at the protection of the pelvic infundibulum from surgical trauma. Interestingly, in a study, 47 the comparison between the excision of dermoid cysts with a mesial approach (33 patients) and an anti-mesial approach (34 patients) showed a higher maintenance of the mean number of antral follicles, a larger mean ovarian diameter, and a higher mean PSV in the ovaries of patients treated with the mesial approach. 47

### Fertility as the Endpoint

Interestingly, only one article 48 addressed the main objective of fertility, translated as the birth rates after ovarian surgery. The follow-up of 60 women for 24 months after ovarian surgery demonstrated a significant decrease in AMH levels (2.7 mcg/l to 1.1 mcg/l; *p* = 0.001). In total, 36 women tried to conceive, 18 became pregnant, and there were 12 live births. It was possible to determine the behavior of the AMH levels in 34 women who attempted to conceive, and it decreased in both groups (pregnant: 3.3 mg/l to 1.0 mg/l; *p* = 0.057; not pregnant: 3.2 mg/l to 2.0 mg/l, *p* = 0.003), but this decrease was not different between the 2 groups ( *p* = 0.112). 48

## Conclusion

We conducted a comprehensive review of the literature for the identification of relevant factors regarding the practical recommendations for the treatment of benign adnexal masses and the insights for fertility preservation. The discussion of the subject is extensive, somewhat controversial, but with some points of convergence. In the menacme, after the diagnosis of an adnexal mass, the incidence of cancer is not high, and the use of magnetic resonance imaging (MRI) and SRs is satisfactory to predict the risk of developing malignancy. Expectant management can be a valid alternative, avoiding unnecessary procedures, surgical complications, and ovarian changes caused by traumatic injuries. The use of anovulatory medications is, in most cases, unnecessary. Dermoid cysts are more associated with torsion, and masses ranging from 8 cm to 15 cm are more common in this kind of complication. Detorsion is a valid option in cases of functional cysts. The use of composite scores of risk factors in the pediatric/adolescent scenario can aid in the decision for surveillance or surgical treatment. When surgery is necessary, some technical aspects are somehow clearer. The use of videolaparoscopy is well established, in which stripping cystectomy shows better results if we consider all types of masses together. Surgery can be performed at any stage of the cycle. Materials should be removed from the abdomen in protective pouches and, preferably, through the umbilical scar incision. Minilaparotomy is acceptable for masses larger than 7 cm. The mesial approach should be considered in cases of teratomas. The use of bipolar electrocautery appears to have an even more negative impact on AMH levels and the AFC after cystectomies, which may persist for 12 months after the surgical procedure, and should be avoided. Suture is preferable. However, caution should be exercised with such an assertion, given the heterogeneity of the available studies. We emphasize the need for a precise standardization of future studies that involve comparing new hemostasis techniques (when to apply them and if they are necessary at all). Specifically in the case of endometriomas, the evidence is unclear, but, apparently, there are no changes in the primary ovulatory function of the affected ovaries, but they may be associated with decreased ovarian reserve caused by the disease itself. In addition, the evidence regarding decreased ovarian reserve after the surgical treatment is very strong. Moreover, in view of the recurrence of endometriomas, the second surgery is even more harmful to the AFC. The precise indication and technical quality of the first surgery is fundamental, and successive surgeries should be considered with great caution. Now, there are no standardized protocols to address endometriomas, especially considering the size as the mark of the decision. In regards to fertility, surveillance seems to be the best alternative. Despite some proposals on this subject, the need for future research is evident. Regarding the ovarian reserve, the current difficulties of using markers that are more reliable are clear. Apparently, estimates may be more accurate when counting the antral follicles (which show the direct impact of the affected ovary) in relation to AMH levels (which reflect the pattern of the two ovaries simultaneously). There is a lack of long-term follow-up studies that can elucidate these differences more clearly. Nevertheless, despite the evidence of the decrease in the parameters of evaluation of the ovarian reserve, the question remains: what is the impact of the adnexal masses and their treatments on the real chances of gestation? The data suggest that ovarian reserve evaluations purely based on AMH levels or the AFC may not satisfactorily reflect the actual risks of infertility. It has been suggested that, although objective assessments of the ovarian reserve are of extreme value, it is necessary to prioritize the focus on long-term studies that present the rate of live births as their endpoint. Therefore, we suggest great caution and care when clarifying the best possible evidence for patients with adnexal masses, revealing our limitations. In this way, we will be able to offer the necessary data about the reproductive future and the adequate information for the correct decision making regarding the patients who need treatment for adnexal pathologies.

## References

[1] Royal College of Obstetricians and Gynaecologists. Management of suspected ovarian masses in premenopausal women London RCOG 2011. (RCOG Green-top Guideline; no. 62)

[2] Lind T Lampic C Hammarström M Rodriguez-Wallberg K Young women's perceptions of fertility-related information and fertility distress before surgery for ovarian cysts Acta Obstet Gynecol Scand 2013 92 11 1290 1296. Doi: 10.1111/aogs.12228 23937414 10.1111/aogs.12228

[3] Jeon J H Park S Y Lee S R Jeong K Chung H W Serum anti-Müllerian hormone levels before surgery in patients with ovarian endometriomas compared to other benign ovarian cysts J Menopausal Med 2015 21 03 142 148. Doi: 10.6118/jmm.2015.21.3.142 26793679 10.6118/jmm.2015.21.3.142 PMC4719088

[4] Lind T Hammarström M Lampic C Rodriguez-Wallberg K Anti-Müllerian hormone reduction after ovarian cyst surgery is dependent on the histological cyst type and preoperative anti-Müllerian hormone levels Acta Obstet Gynecol Scand 2015 94 02 183 190. Doi: 10.1111/aogs.12526 25287421 10.1111/aogs.12526

[5] Uncu G Kasapoglu I Ozerkan K Seyhan A Oral Yilmaztepe A Ata B Prospective assessment of the impact of endometriomas and their removal on ovarian reserve and determinants of the rate of decline in ovarian reserve Hum Reprod 2013 28 08 2140 2145. Doi: 10.1093/humrep/det123 23624580 10.1093/humrep/det123

[6] Mohamed A A Al-Hussaini T K Fathalla M M El Shamy T T Abdelaal I I Amer S A The impact of excision of benign nonendometriotic ovarian cysts on ovarian reserve: a systematic review Am J Obstet Gynecol 2016 215 02 169 176. Doi: 10.1016/j.ajog.2016.03.045 27059508 10.1016/j.ajog.2016.03.045

[7] Grimes D A Jones L B Lopez L M Schulz K F Oral contraceptives for functional ovarian cysts Cochrane Database Syst Rev 2014 04 CD006134. Doi: 10.1002/14651858.CD006134.pub5 17054275 10.1002/14651858.CD006134.pub2

[8] Hermans A J Kluivers K B Janssen L M Siebers A G Wijnen M HWA Bulten J Adnexal masses in children, adolescents and women of reproductive age in the Netherlands: A nationwide population-based cohort study Gynecol Oncol 2016 143 01 93 97. Doi: 10.1016/j.ygyno.2016.07.096 27421754 10.1016/j.ygyno.2016.07.096

[9] Azarakhsh N Grimes S Chotai P N Shephard C Huang E Y Post-resection outcomes for pediatric ovarian neoplasm: is ovarian-preserving surgery a good option? Pediatr Surg Int 2017 33 01 97 104. Doi: 10.1007/s00383-016-3987-x 27738824 10.1007/s00383-016-3987-x

[10] Reid B M Permuth J B Sellers T A Epidemiology of ovarian cancer: a review Cancer Biol Med 2017 14 01 9 32. Doi: 10.20892/j.issn.2095-3941.2016.0084 28443200 10.20892/j.issn.2095-3941.2016.0084 PMC5365187

[11] Kaijser J Sayasneh A Van Hoorde K Ghaem-Maghami S Bourne T Timmerman D Van Calster B Presurgical diagnosis of adnexal tumours using mathematical models and scoring systems: a systematic review and meta-analysis Hum Reprod Update 2014 20 03 449 462. Doi: 10.1093/humupd/dmt059 24327552 10.1093/humupd/dmt059

[12] Timmerman D Testa A C Bourne T Ameye L Jurkovic D Van Holsbeke C Simple ultrasound-based rules for the diagnosis of ovarian cancer Ultrasound Obstet Gynecol 2008 31 06 681 690. Doi: 10.1002/uog.5365 18504770 10.1002/uog.5365

[13] Froyman W Wynants L Landolfo C Bourne T Valentin L Testa A Validation of the performance of International Ovarian Tumor Analysis (IOTA) methods in the diagnosis of early stage ovarian cancer in a non-screening population Diagnostics (Basel) 2017 7 02 E32. Doi: 10.3390/diagnostics7020032 10.3390/diagnostics7020032 PMC5489952 28574444

[14] Oktay K Harvey B E Partridge A H Bourne T Valentin L Testa A Fertility preservation in patients with cancer: ASCO clinical practice guideline update J Clin Oncol 2018 36 19 1994 2001. Doi: 10.1200/JCO.2018.78.1914 29620997 10.1200/JCO.2018.78.1914

[15] Fleming R Seifer D B Frattarelli J L Ruman J Assessing ovarian response: antral follicle count versus anti-Müllerian hormone Reprod Biomed Online 2015 31 04 486 496. Doi: 10.1016/j.rbmo.2015.06.015 26283017 10.1016/j.rbmo.2015.06.015

[16] Alson S SE Bungum L J Giwercman A Henic E Anti-müllerian hormone levels are associated with live birth rates in ART, but the predictive ability of anti-müllerian hormone is modest Eur J Obstet Gynecol Reprod Biol 2018 225 199 204. Doi: 10.1016/j.ejogrb.2018.04.039 29738982 10.1016/j.ejogrb.2018.04.039

[17] Muzii L Di Tucci C Di Feliciantonio M Marchetti C Perniola G Panici P B The effect of surgery for endometrioma on ovarian reserve evaluated by antral follicle count: a systematic review and meta-analysis Hum Reprod 2014 29 10 2190 2198. Doi: 10.1093/humrep/deu199 25085800 10.1093/humrep/deu199

[18] Alebić MŠ Stojanović N Dewailly D Discordance between serum anti-Müllerian hormone concentrations and antral follicle counts: not only technical issues Hum Reprod 2018 33 06 1141 1148. Doi: 10.1093/humrep/dey098 29688494 10.1093/humrep/dey098

[19] Committee on Gynecologic Practice.Committee opinion no. 618: Ovarian reserve testing Obstet Gynecol 2015 125 01 268 273 10.1097/01.AOG.0000459864.68372.ec 25560143

[20] Froyman W Landolfo C De Cock B Wynants L Sladkevicius P Testa A C Risk of complications in patients with conservatively managed ovarian tumours (IOTA5): a 2-year interim analysis of a multicentre, prospective, cohort study Lancet Oncol 2019 20 03 448 458. Doi: 10.1016/S1470-2045(18)30837-4 30737137 10.1016/S1470-2045(18)30837-4

[21] Balci O Energin H Görkemli H Acar A Management of adnexal torsion: a 13-year experience in single tertiary center J Laparoendosc Adv Surg Tech A 2019 29 03 293 297. Doi: 10.1089/lap.2018.0307 30118383 10.1089/lap.2018.0307

[22] Schwartz B I Huppert J S Chen C Huang B Reed J L Creation of a composite score to predict adnexal torsion in children and adolescents J Pediatr Adolesc Gynecol 2018 31 02 132 137. Doi: 10.1016/j.jpag.2017.08.007 28847757 10.1016/j.jpag.2017.08.007

[23] Leone Roberti Maggiore U Scala C Venturini P L Remorgida V Ferrero S Endometriotic ovarian cysts do not negatively affect the rate of spontaneous ovulation Hum Reprod 2015 30 02 299 307. Doi: 10.1093/humrep/deu308 25432923 10.1093/humrep/deu308

[24] Somigliana E Benaglia L Paffoni A Busnelli A Vigano P Vercellini P Risks of conservative management in women with ovarian endometriomas undergoing IVF Hum Reprod Update 2015 21 04 486 499. Doi: 10.1093/humupd/dmv012 25750209 10.1093/humupd/dmv012

[25] Legendre G Catala L Morinière C Lacoeuille C Boussion F Sentilhes L Descamps P Relationship between ovarian cysts and infertility: what surgery and when? Fertil Steril 2014 101 03 608 614. Doi: 10.1016/j.fertnstert.2014.01.021 24559614 10.1016/j.fertnstert.2014.01.021

[26] Hart R Hickey M Maouris P Buckett W Garry R Excisional surgery versus ablative surgery for ovarian endometriomata: a Cochrane Review Hum Reprod 2005 20 11 3000 3007. Doi: 10.1093/humrep/dei207 16246860 10.1093/humrep/dei207

[27] Ding Y Yuan Y Ding J Chen Y Zhang X Hua K Comprehensive assessment of the impact of laparoscopic ovarian cystectomy on ovarian reserve J Minim Invasive Gynecol 2015 22 07 1252 1259. Doi: 10.1016/j.jmig.2015.07.011 26210677 10.1016/j.jmig.2015.07.011

[28] Kwon S K Kim S H Yun S C Kim D Y Chae H D Kim C H Kang B M Decline of serum antimüllerian hormone levels after laparoscopic ovarian cystectomy in endometrioma and other benign cysts: a prospective cohort study Fertil Steril 2014 101 02 435 441. Doi: 10.1016/j.fertnstert.2013.10.043 24290000 10.1016/j.fertnstert.2013.10.043

[29] Cagnacci A Bellafronte M Xholli A Palma F Carbone M M Di Carlo C Grandi G Impact of laparoscopic cystectomy of endometriotic and non-endometriotic cysts on ovarian volume, antral follicle count (AFC) and ovarian doppler velocimetry Gynecol Endocrinol 2016 32 04 298 301. Doi: 10.3109/09513590.2016.1142523 26850447 10.3109/09513590.2016.1142523

[30] Chen Y Pei H Chang Y Chen M Wang H Xie H Yao S The impact of endometrioma and laparoscopic cystectomy on ovarian reserve and the exploration of related factors assessed by serum anti-Mullerian hormone: a prospective cohort study J Ovarian Res 2014 7 108. Doi: 10.1186/s13048-014-0108-0 25424986 10.1186/s13048-014-0108-0 PMC4255637

[31] Jang W K Lim S Y Park J C Lee K R Lee A Rhee J H Surgical impact on serum anti-Müllerian hormone in women with benign ovarian cyst: A prospective study Obstet Gynecol Sci 2014 57 02 121 127. Doi: 10.5468/ogs.2014.57.2.121 24678485 10.5468/ogs.2014.57.2.121 PMC3965695

[32] Kim Y J Cha S W Kim H O Serum anti-Müllerian hormone levels decrease after endometriosis surgery J Obstet Gynaecol 2017 37 03 342 346. Doi: 10.1080/01443615.2016.1239071 28129715 10.1080/01443615.2016.1239071

[33] Rustamov O Krishnan M Roberts S A Fitzgerald C T Effect of salpingectomy, ovarian cystectomy and unilateral salpingo-oopherectomy on ovarian reserve Gynecol Surg 2016 13 173 178. Doi: 10.1007/s10397-016-0940-x 27478428 10.1007/s10397-016-0940-x PMC4949297

[34] Muzii L Di Tucci C Di Feliciantonio M Galati G Pecorella I Radicioni A Ovarian reserve reduction with surgery is not correlated with the amount of ovarian tissue inadvertently excised at laparoscopic surgery for endometriomas Reprod Sci 2019 26 11 1493 1498. Doi: 10.1177/1933719119828055 30764716 10.1177/1933719119828055

[35] Rodriguez-Purata J Gonzalez-Foruria I Montoya-Botero P Rodriguez I Hereter L Polyzos N P Ultrasonographically diagnosed dermoid cysts do not influence ovarian stimulation response in an *in vitro* fertilization cycle Gynecol Endocrinol 2019 35 07 612 617. Doi: 10.1080/09513590.2018.1563887 30727778 10.1080/09513590.2018.1563887

[36] Var T Batioglu S Tonguc E Kahyaoglu I The effect of laparoscopic ovarian cystectomy versus coagulation in bilateral endometriomas on ovarian reserve as determined by antral follicle count and ovarian volume: a prospective randomized study Fertil Steril 2011 95 07 2247 2250. Doi: 10.1016/j.fertnstert.2011.03.078 21481381 10.1016/j.fertnstert.2011.03.078

[37] Litta P D'Agostino G Conte L Saccardi C Cela V Angioni S Plebani M Anti-Müllerian hormone trend after laparoscopic surgery in women with ovarian endometrioma Gynecol Endocrinol 2013 29 05 452 454. Doi: 10.3109/09513590.2012.758704 23368705 10.3109/09513590.2012.758704

[38] Saito N Okuda K Yuguchi H Yamashita Y Terai Y Ohmichi M Compared with cystectomy, is ovarian vaporization of endometriotic cysts truly more effective in maintaining ovarian reserve? J Minim Invasive Gynecol 2014 21 05 804 810. Doi: 10.1016/j.jmig.2014.03.008 24681062 10.1016/j.jmig.2014.03.008

[39] Nappi L Angioni S Sorrentino F Cinnella G Lombardi M Greco P Anti-Mullerian hormone trend evaluation after laparoscopic surgery of monolateral endometrioma using a new dual wavelengths laser system (DWLS) for hemostasis Gynecol Endocrinol 2016 32 01 34 37. Doi: 10.3109/09513590.2015.1068754 26359914 10.3109/09513590.2015.1068754

[40] Kang J H Kim Y S Lee S H Kim W Y Comparison of hemostatic sealants on ovarian reserve during laparoscopic ovarian cystectomy Eur J Obstet Gynecol Reprod Biol 2015 194 64 67. Doi: 10.1016/j.ejogrb.2015.08.010 26344349 10.1016/j.ejogrb.2015.08.010

[41] Zhang C H Wu L Li P Q Clinical study of the impact on ovarian reserve by different hemostasis methods in laparoscopic cystectomy for ovarian endometrioma Taiwan J Obstet Gynecol 2016 55 04 507 511. Doi: 10.1016/j.tjog.2015.08.026 27590372 10.1016/j.tjog.2015.08.026

[42] Deckers P Ribeiro S C Simões R DS Miyahara C BDF Baracat E C Systematic review and meta-analysis of the effect of bipolar electrocoagulation during laparoscopic ovarian endometrioma stripping on ovarian reserve Int J Gynaecol Obstet 2018 140 01 11 17. Doi: 10.1002/ijgo.12338 28980317 10.1002/ijgo.12338

[43] Romualdi D Franco Zannoni G Lanzone A Selvaggi L Tagliaferri V Gaetano Vellone V Follicular loss in endoscopic surgery for ovarian endometriosis: quantitative and qualitative observations Fertil Steril 2011 96 02 374 378. Doi: 10.1016/j.fertnstert.2011.05.078 21703608 10.1016/j.fertnstert.2011.05.078

[44] Raffi F Metwally M Amer S The impact of excision of ovarian endometrioma on ovarian reserve: a systematic review and meta-analysis J Clin Endocrinol Metab 2012 97 09 3146 3154. Doi: 10.1210/jc.2012-1558 22723324 10.1210/jc.2012-1558

[45] Somigliana E Berlanda N Benaglia L Viganò P Vercellini P Fedele L Surgical excision of endometriomas and ovarian reserve: a systematic review on serum antimüllerian hormone level modifications Fertil Steril 2012 98 06 1531 1538. Doi: 10.1016/j.fertnstert.2012.08.009 22975114 10.1016/j.fertnstert.2012.08.009

[46] Song T Kim M K Kim M L Jung Y W Yun B S Seong S J Effect of menstrual phase on the surgical treatment of ovarian cysts J Obstet Gynaecol 2017 37 07 919 923. Doi: 10.1080/01443615.2017.1312313 28597715 10.1080/01443615.2017.1312313

[47] Morelli M Mocciaro R Venturella R Imperatore A Lico D Zullo F Mesial side ovarian incision for laparoscopic dermoid cystectomy: a safe and ovarian tissue-preserving technique Fertil Steril 2012 98 05 1336 400. Doi: 10.1016/j.fertnstert.2012.07.1112 22884658 10.1016/j.fertnstert.2012.07.1112

[48] Lind T Lampic C Olofsson J I Rodriguez-Wallberg K A Postoperative AMH reduction is not associated with reduced fecundity two years following ovarian cyst surgery Gynecol Endocrinol 2016 32 09 745 748. Doi: 10.3109/09513590.2016.1166198 27028572 10.3109/09513590.2016.1166198

